# Accurate and fast mitotic detection using an anchor-free method based on full-scale connection with recurrent deep layer aggregation in 4D microscopy images

**DOI:** 10.1186/s12859-021-04014-w

**Published:** 2021-02-26

**Authors:** Titinunt Kitrungrotsakul, Yutaro Iwamoto, Satoko Takemoto, Hideo Yokota, Sari Ipponjima, Tomomi Nemoto, Lanfen Lin, Ruofeng Tong, Jingsong Li, Yen-Wei Chen

**Affiliations:** 1 Research Center for Healthcare Data Science, Zhejiang Lab, Hangzhou, China; 2 Graduate School of Information Science and Engineering, Ritsumeikan University, Kusatsu, Shiga Japan; 3 Center for Advanced Photonics, RIKEN, Wako, Saitama Japan; 4 Research Institute for Electronic Science, Hokkaido University, Sapporo, Hokkaido Japan; 5 College of Computer Science and Technology, Zhejiang University, Hangzhou, China; 6 College of Biomedical Engineering and Instrument Science, Zhejiang University, Hangzhou, China

**Keywords:** 4D image, cell, detection, microscopic image, mitotic

## Abstract

**Background:**

To effectively detect and investigate various cell-related diseases, it is essential to understand cell behaviour. The ability to detection mitotic cells is a fundamental step in diagnosing cell-related diseases. Convolutional neural networks (CNNs) have been successfully applied to object detection tasks, however, when applied to mitotic cell detection, most existing methods generate high false-positive rates due to the complex characteristics that differentiate normal cells from mitotic cells. Cell size and orientation variations in each stage make detecting mitotic cells difficult in 2D approaches. Therefore, effective extraction of the spatial and temporal features from mitotic data is an important and challenging task. The computational time required for detection is another major concern for mitotic detection in 4D microscopic images.

**Results:**

In this paper, we propose a backbone feature extraction network named full scale connected recurrent deep layer aggregation (RDLA++) for anchor-free mitotic detection. We utilize a 2.5D method that includes 3D spatial information extracted from several 2D images from neighbouring slices that form a multi-stream input.

**Conclusions:**

Our proposed technique addresses the scale variation problem and can efficiently extract spatial and temporal features from 4D microscopic images, resulting in improved detection accuracy and reduced computation time compared with those of other state-of-the-art methods.

## Background

Mitosis is the process by which a cell divides itself into two identical cells [1]. Observing and analysing cell behaviours is advantageous in multiple applications, such as predicting breast cancer, drug discovery, identifying stem cells, and developing abnormal skin structures. The conventional techniques for detecting and counting mitotic cells are performed manually by specialists.

Mitotic cells are detected and counted by observing a sample preserved between glass slides under a microscope [2–4]. While three-dimensional (3D) images are normally involved, instead, a sequence of 2D images is captured at different times. Although various methods have been proposed to solve mitotic cell detection problems [5, 6], a cell may freely perform mitosis in any orientation. Thus, capturing mitotic cells in 2D images may lead to a loss of spatial features due to different cell orientations.

**Fig. 1:**
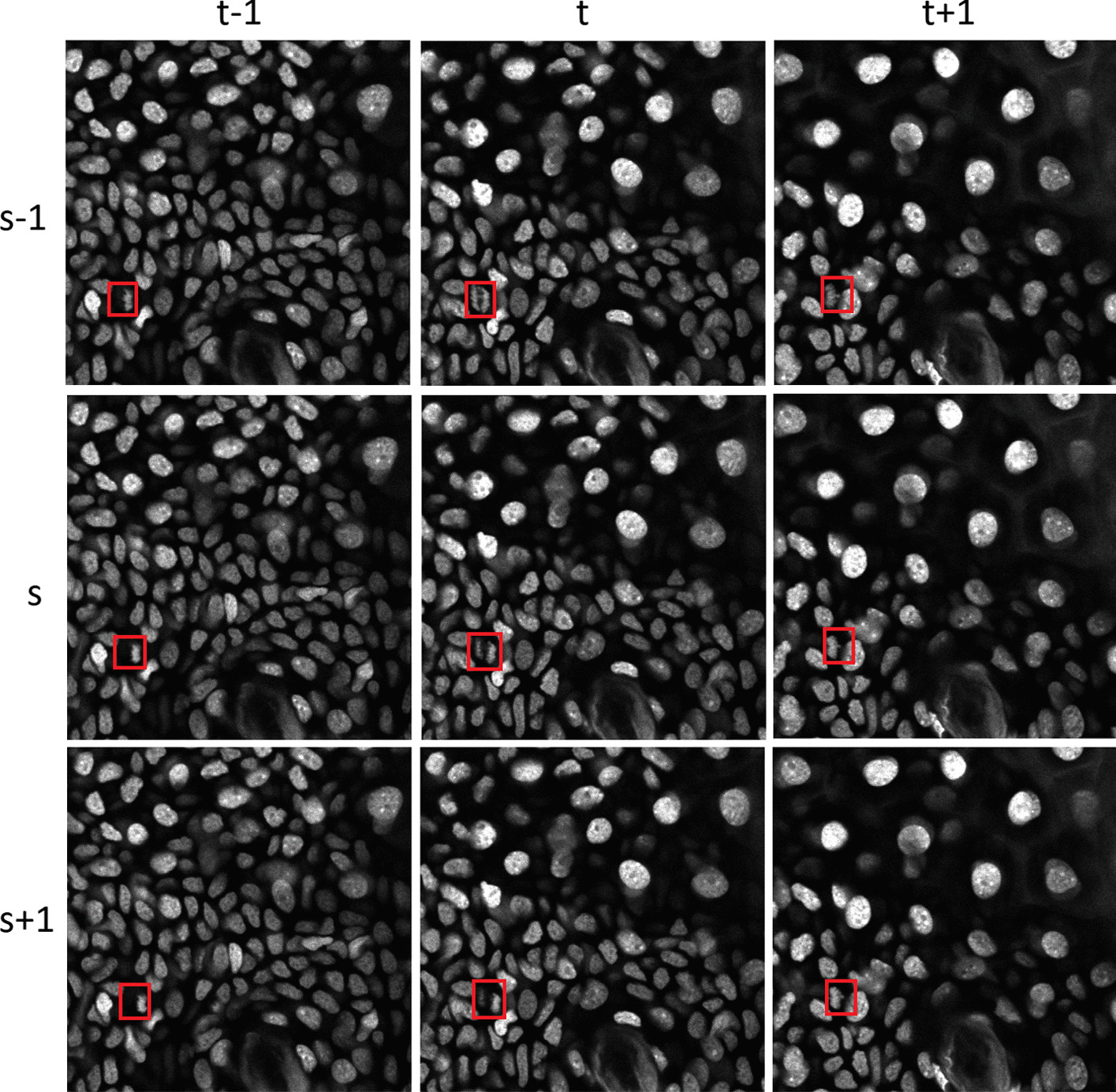
Samples of three neighbouring slices ($s-1, s, s+1$) of a mitotic event at different time periods ($t-1, t, t+1$). The red boxes indicate mitotic events

Because cell orientation is critically important for determining various cell types during developmental periods [7, 8], the two-photon microscope was proposed as an alternative to manually examining samples in glass slides for epidermal imaging [9, 10]. This examination method is utilized to capture 4D data (time sequences of 3D images) and analyse cellular behaviour. Skin diseases, such as cancer, ichthyosis vulgaris, atopic dermatitis, and abnormal skin structures, can be predicted from a comprehensive analysis of cellular behaviour [11–13]. Augmenting the information by using 4D data reduces the resources and time needed to detect mitotic cells but also increases the required effort. Fig. 1 depicts cell images at various slice indexes $\{s-1,s,s+1\}$ (spatial information) and time frames $\{t-1,t,t+1\}$ (temporal information) from a 4D microscopic image (the mitotic cells are indicated by the bounding boxes).

Manually detecting mitotic cells in 4D microscopic images is a labour-and time-intensive task, which makes the ability to perform automatic cell detection in 4D microscopic images desirable. Although several automatic methods have been proposed for mitotic cell detection [5, 6, 14, 15], the existing challenges are as follows: First, mitotic cells can be grouped into several stages from prophase to mitosis, and cell size varies drastically among these stages. We need to develop a scale-invariant detection method to address scale variance, which dramatically affects mitotic detection. Second, the cells may divide while oriented in any direction, and the direction may change over time. Therefore, we need an orientation-robust method that considers 3D spatial and temporal information. Third, to detect mitotic events in 4D images while reducing the computational time, we need an efficient and fast detection method.

Recently, high-performance object detection has been developed utilizing convolutional neural network (CNN) models. These object detection networks can be categorized into two classes, anchor-based and anchor-free networks, based on their detection procedure. Numerous anchor-based detection approaches, such as single-shot multibox detectors (SSDs) [16], Faster R-CNN [17], YOLO [18], feature pyramid networks (FPNs) [19], and Mask RCNN [20], have been proposed for object detection in natural images. In our previous work, we developed anchor-based detection techniques for 4D microscopic images and achieved improved detection performances compared to other existing anchor-based methods [14, 15]. However, anchor-based methods have lengthy runtimes (although the computation time can be reduced by running the algorithms on multiple GPUs), and they still produce false negatives due to scale and orientation problems [15]. Fine-tuning of anchor-based detector models usually requires hyperparameter tuning, which is a critical step that affects the network’s performance.

In addition, anchor-free detection approaches have been recently proposed, including CornerNet [21], ExtremeNet [22], and CenterNet ( objects as points) [23] and achieve superior performances over anchor-based approaches. They also ameliorate the problems of hyperparameter tuning and lengthy computational times.

Anchor-based and anchor-free detection methods both consist of two parts: a feature extractor backbone network and a detection head. In this paper, we concentrated on improving the feature extractor for object detection to address the scale variation problem. We propose a full-scale connected recurrent deep layer aggregation network to extract effective full-scale spatial and temporal information for mitotic basal epidermal cell detection from 4D microscope data. The proposed technique includes two main parts. The first is a full-scale connected deep layer aggregation network (DLA++), which is an improved version of the existing deep layer aggregation (DLA) model [24]. The proposed DLA++ converts low-level features to high-level features, including the scale information, while avoiding the loss of useful information. The second is a recurrent DLA++ (RDLA++), to which we added a convolutional long short-term memory (CLSTM) model to DLA++. This module extracts temporal information and reduces the number of false positives. Moreover, to reduce the number of false negatives, we use a 2.5D technique that extracts 3D information from a set of 2D images sourced from neighbouring slices, forming a multistream input that includes 3D spatial information [14]. To achieve accurate and fast mitotic detection, we combine the proposed RDLA++ with anchor-free detection heads (i.e., CenterNet). The proposed method includes three main contributions. First, we propose a scale-insensitive anchor-free detection method for solving the scale variation problem and to perform mitotic cell detection of various sizes. Second, we propose an orientation-robust 2.5D recurrent model to extract full spatial and temporal features that enable accurate detection of 3D mitotic cells dividing in any direction. Third, we combine the proposed RDLA++ with anchor-free detection heads (CenterNet) to obtain a fast and accurate mitotic detection algorithm. Our experimental results show that the proposed techniques achieve better performances than do other compared state-of-the-art techniques.

## Related works

### Detecting objects through deep learning

Deep learning methods have achieved state-of-the-art results in object detection and can be grouped into anchor-based and anchor-free methods.

Region proposal networks (RPNs) were first proposed as a part of Faster R-CNN [17] and are the concept underlying most anchor-based object detection, including SSDs [16] and Mask RCNN [20]. RPNs involve three main processes. The first is feature extraction, in which a CNN transforms an input image into high-level feature maps. The second process involves creating candidate bounding boxes using a set of predefined anchors to extract candidate objects from the feature maps. Nine anchors of three different aspect ratios with three scales are commonly used in RPNs. The final process involves classification and regression of the candidate bounding boxes. The main problem is the vanishing features related to small objects in RPNs, which degrades RPN performances for smaller objects.

Recently, anchor-free detection approaches have been proposed that outperform the anchor-based methods. The anchor-free detection technique also addresses the problem of tuning the anchor hyperparameters in anchor-based approaches. CornerNet [21] is an anchor-free object detection method proposed by H. Law et al., who found that the detection results can be reconstructed using the corner points of the bounding boxes. Their network can be regarded as the first one-stage object detection method, and it surpassed the performances of two-stage object detectors such as Faster-RCNN regarding accuracy and computation time. An improved version of CornerNet, called ExtremeNet [22], was proposed by X. Zhou et al. ExtremeNet attempted to solve the bounding reconstruction problem in CornerNet. The authors proposed using a centre point and most extreme points created by their network to create bounding boxes. ExtremeNet can be combined with the deep extreme cut (DEXTR) algorithm [25] to conduct segmentation tasks. X. Zhou proposed CenterNet (object as points) [23] in which a detection head was proposed that could work with various networks, such as residual networks (ResNets) [26], hourglass networks (HourglassNets) [27], and deep layer aggregation (DLA) [24]. CenterNet detects objects as centre points; then, the size (height and width) of each object’s bounding box is determined through regression. However, both approaches are designed to perform detection on normal images; none of the existing anchor-free networks concentrate on mitotic detection tasks.

### Mitotic detection methods

The use of binarization [28] or segmentation methods [29] has been proposed for the traditional detection methods of mitotic detection. Both methods are nondeep learning methods, thus they do not require large amounts of data to obtain higher detection accuracy. However, they require time-consuming alignment methods to obtain high detection performances.

Mao et al. (2016, 2017) proposed a hierarchical convolutional neural network (HCNN) [6] and a two-stream bidirectional long short-term memory (TS-BLSTM) model [5] to detect and identify mitotic cells, respectively. Both methods accept two types of images (appearance and motion images) as input. To solve the problem of HCNN, the authors proposed using LSTM in TS-BLSTM to extract temporal features. The performance of TS-BLSTM was significantly improved compared to that of HCNN. However, both techniques were suitable only for mitotic cell detection from 3D microscopic images (time sequences of 2D images). These methods do not include spatial information when predicting the detection results.

Kitrungrotsakul et al. [14, 15] presented a 2.5D mitotic cell detection method using a CLSTM [30] to detect mitotic cells in 4D microscopic images (time sequences of 3D images). They utilized three slices (a target slice and its neighbouring slices) as input images, known as 2.5D input, to obtain 3D spatial features and enhance the detection accuracy. However, this method still has difficulties because it omits cells not initiating mitosis within the captured image. These cells were divided into two daughter cells around the image boundary.

Both Mao and Kitrungrotsakul focused on anchor-based detection techniques, which require lengthy computational times and a preparation step to tune the anchor-hyperparameters.

## Design and implementation

**Fig. 2:**
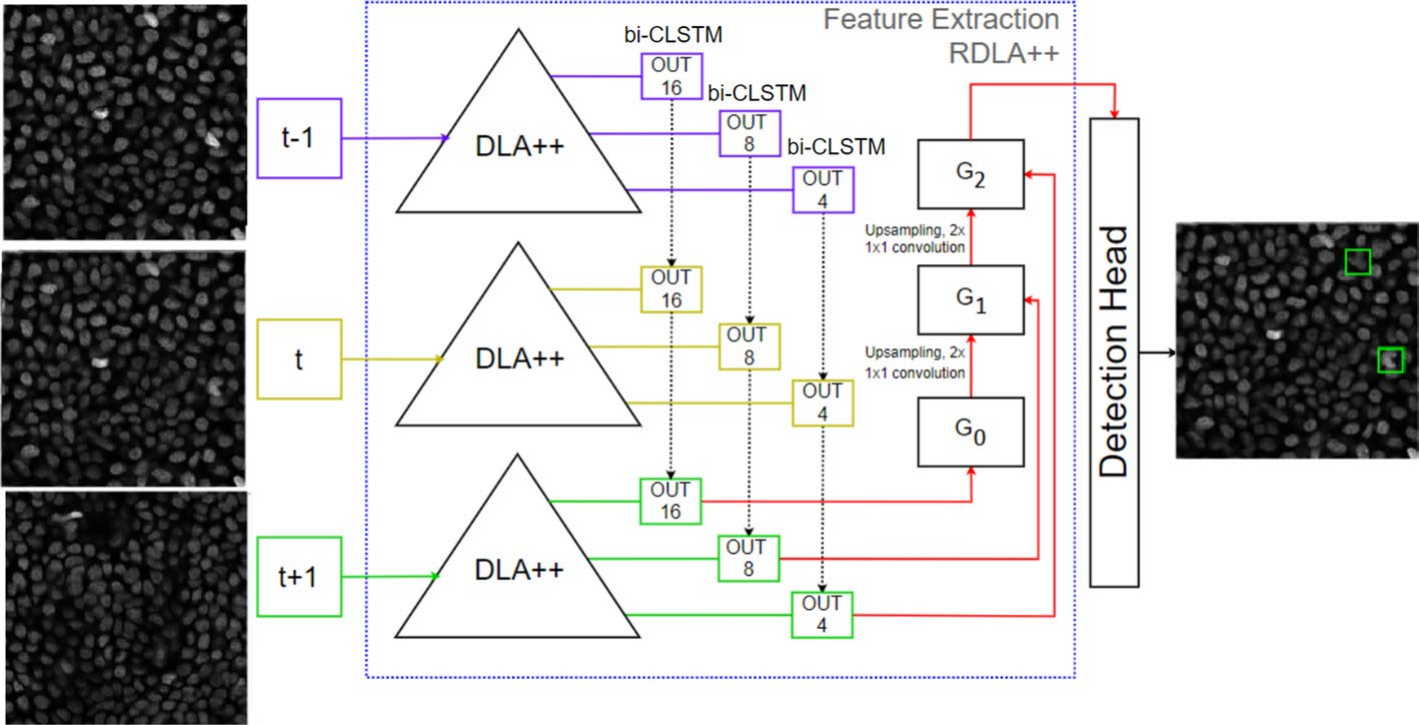
An architectural overview of RDLA++. The blue box indicates our proposed backbone RDLA++ for spatial and temporal feature extraction in 4D microscopy images. DLA++ depicts our proposed full-scale connected deep layer aggregation network, which is an enhanced version of DLA. The numbers 4, 8, and 16 represent scale factors (for example, 4 denotes the original size downsampled to a size of 1/4). The smaller number indicates lower-level features. The spatial features extracted from various time frames ($t-1, t, t+1$) by DLA++ are fed into a convolution long short-term memory (CLSTM) for temporal feature extraction. G denotes the upsampling operations and 1$\times$1 convolutions are adopted to preserve and use the low-level features in the final decision. The final combined temporal and spatial features are input to the detection head (CenterNet). Note that the proposed RDLA++ can be integrated with any anchor-free detection header

Figure  2 depicts the network architecture of our proposed detection network. In this study, we focus primarily on feature manipulation and feature extraction; any anchor-free detection head can be utilized, including CenterNet, CornerNet, or others.

### Full-scale connected deep layer aggregation network (DLA++)

In DLA++, the linear skip connection in the original DLA is replaced by the hierarchical skip connection concept. The DLA network was designed to solve problems that occurred with other types of skip connections, such as those in FCN [31], FPN [19], and U-NET [32] by using a linear skip connection to pass same-scale features from lower to upper layers. Nevertheless, some feature information is lost in the networks after each sequential hierarchy level. To preserve the information and reutilize full-scale features, we applied the concept from DenseNet [33] to the DLA model and present a full-scale connected deep layer aggregation (DLA++).

**Fig. 3:**
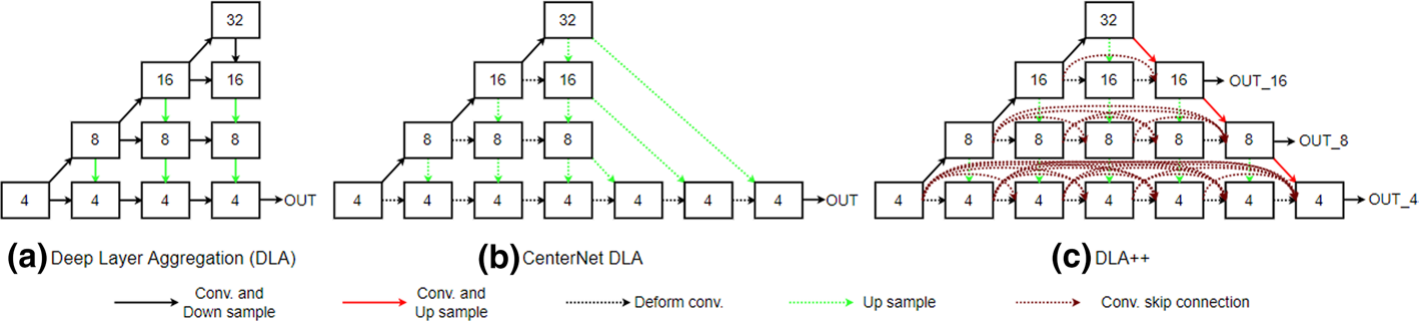
A comparison of **a** DLA, **b** the DLA of CenterNet, and **c** the DLA++ in our proposed network. The numbers 4, 8, 16, and 32 in each box denote scale factors that represent the feature map sizes (smaller numbers indicate lower-level features, and larger numbers indicate higher-level features). The proposed DLA++ represents full-scale skip connections, which incorporate low-level details with high-level semantics from feature maps at full scales. Three features from different levels (Out 4, Out 8, and Out 16) are output to detect mitotic events

The proposed DLA++ model (Fig. 3.c) was inspired by both DenseNet and DLA. In Fig. 3, the numbers 4, 8, 16, and 32 denote scale factors that represent the changes in the spatial dimension of each feature map (for instance, a 4 represents the original feature size downsampled to a size of 1/4). Smaller numbers indicate lower-level features, and larger numbers represent higher-level features. The proposed DLA++ generates features of three different scales (Out 4, Out 8, Out 16) for mitotic detection.

As shown in Fig. 3, the proposed DLA++ integrates multiscale features by designing dense skip connections to pass lower- to higher-level features as well as passing lower features from the upper-node to other upper-node-level features at the same level. The full-scale-level features are then utilized for mitotic detection. We use ${x^i_j}$ to denote the output from node *X*, in which *i* is the level index of the downsampling layer and *j* represents the deformable convolutional dense layer along with the skip connection, where *J* denotes the number of dense layers,

1 $$\begin{aligned} {x^i_j} = \left\{ \begin{array}{ll} H([x^{i-1}_j]), &\quad {\hbox {j}} = 0 \\ H([U(x^{i+1}_{j-1}),[x^i_k]_{k=0}^{j-1}]), &\quad 0< j < J-((i\times 2)+1) \\ H([U(x^{i+1}_{j-2}),[x^i_k]_{k=0}^{j-1}]), &\quad {\hbox {otherwise}} \\ \end{array} \right. \end{aligned}$$

and $H(\cdot )$ denotes a set of functions as a convolutional operation followed by ReLU activation. $U(\cdot )$ denotes an upsampling operation, and $[\cdot ]$ represents a concatenation-layer function. In the equation, at least two inputs are received by all nodes at each *i* level from the deformable convolution layer and the same *i* level as well as upsampling from the upper level $(i+1)$. However, the nodes at each dense level $j = 0$ receive only one input. In addition to the two inputs from deformable convolution and upsampling, other feature inputs are received by all nodes where $j > 0$ from the previous nodes at the same level *i* since these input features indicate a dense level skip connection (DLA++). The reutilization of features in DLA++ reduces the number of network parameters and constitutes an efficient way to improve network performance.

### Recurrent DLA++ (RDLA++)

As explained in the DLA++ section, the proposed DLA++ effectively extracts full-scale features to achieve scale-insensitive object detection (2D mitotic cell detection). To solve the orientation problem in mitotic cell detection, in addition to DLA++, we propose recurrent DLA++ (RDLA++). RDLA++ extracts spatial and temporal features from 4D microscopic images, resulting in accurate and orientation-robust 3D mitotic cell detection. The multistream concept is utilized to form a 2.5D network that extracts spatial information, as explained in previous works [14, 15]. The CLSTM is utilized to extract temporal features from the 4D microscopic image at time *t* from each level *i* in the DLA++ network. Upsampling and 1$\times$1 convolution are used to preserve these features; then, these lower-level features are used in the final decision process. Using both spatial and temporal features, we can obtain features with the same shape and extract multiscale features. We use $\hat{x}^s$ to denote the output from node $G^s$, where *s* represents the indexes of the scale layer,

2 $$\begin{aligned} {\hat{x}^s} = \left\{ \begin{array}{ll} \begin{aligned} H_{1\times 1}(C(x^{I-1}_{J-((i\times 2)+1),0},x^{I-1}_{J-((i\times 2)+1),\ldots }, \\ x^{I-1}_{J-((i\times 2)+1),m})), \end{aligned} &{} {\hbox {s}} = 0 \\ \begin{aligned} H_{1\times 1}([C(x^{I-(s+1)}_{J-((i\times 2)+1),0},x^{I-(s+1)}_{J-((i\times 2)+1),\ldots },\\ x^{I-(s+1)}_{J-((i\times 2)+1),m}), [\hat{x}^l]_{l=0}^{s-1}]), \end{aligned} &{} {\hbox {otherwise}} \\ \end{array} \right. \end{aligned}$$

$C(\cdot )$ represents the convolution LSTM operation on the outputs of the DLA++ network on the microscopic image (*m*) and index level *i*, *I* indicates the number of network levels, and $H_{1\times 1}$ denotes the upsampling operation by a $1\times 1$ convolutional layer.

### The refinement for 4D cell detection

**Fig. 4:**
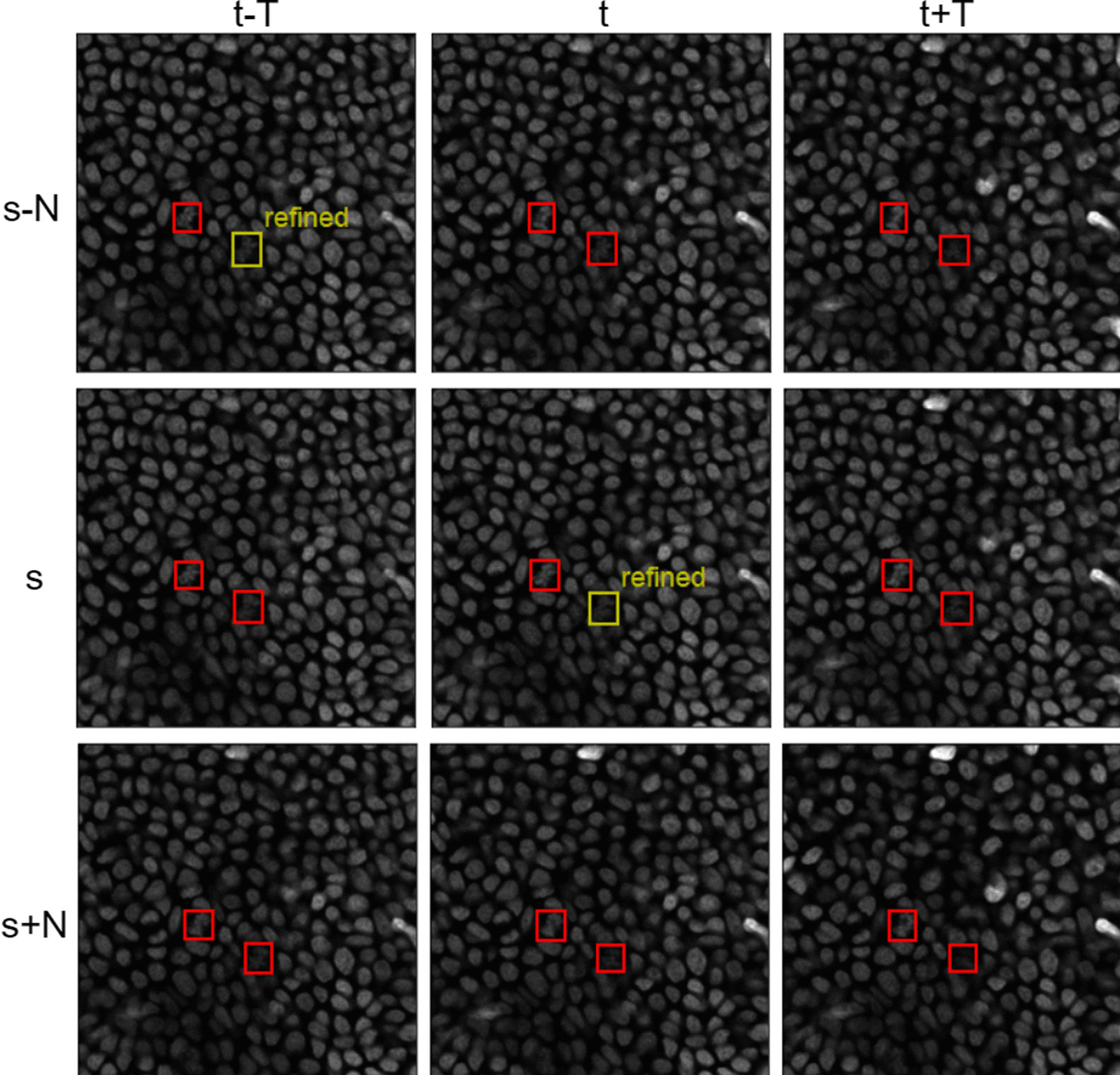
The 4D bounding box refinement. The red boxes represent output bounding boxes from the network. The yellow boxes indicate refinement bounding boxes created by Eq. (5)

This work mainly aimed at performing mitotic detection on 4D microscopic images. We attach an anchor-free detection head such as CenterNet or CornerNet to our backbone network as discussed in the previous section (see Fig. 2). In this study, we consider only bounding boxes ($B_{s,i}^t=<p,x,y,w,h>$) because the results vary in the ultimate outputs based on the detection head. The p represents the prediction of bounding box *i*, where the point *x*, *y* denotes its location, and *w* and *h* denote its width and height, respectively, at slice index *s* and time sequence *t*.

Due to the high similarity between a temporal frame $(t \pm k)$ and the neighbouring spatial slice $(s \pm j)$ of each slice, we calculate a refined value of the predicted *p* of each bounding box from its neighbours as follows:

3 $$\begin{aligned} \hat{p}_{s+j,i}^{t+k} = {\left\{ \begin{array}{ll} 1, &{} \text {if } p_{s+j,i}^{t+k} \ge 0.5\\ 0, &{} \text {else} \end{array}\right. } \end{aligned}$$

4 $$\begin{aligned} W_{s+j,i}^{t+k} =1 - \frac{\sqrt{j^2+k^2}}{\sqrt{N^2+T^2}} \end{aligned}$$

5 $$\begin{aligned} \hat{\hat{p}}_{s+j,i}^{t+k} = \frac{\sum _{j=-N}^N \sum _{k=-T}^T W_{s+j,i}^{t+k} \hat{p}_{s+j,i}^{t+k}}{\sum _{j=-N}^N \sum _{k=-T}^T W_{s+j,i}^{t+k}} \end{aligned}$$

where $\hat{\hat{p}}_{s,i}^t$ denotes the refined value of the prediction at bounding $B_{s,i}^t$ and *W* represents a weighted distance between the neighbouring and target slices: the greater the distance is, the lighter the weight is. *N* denotes the number of neighbouring slices (spatially), and *T* represents the temporal slices (Fig. 4). In this experiment, we set *N* equal to 4 and *T* equal to 6 to enhance the detection results.

## Results

### Experimental setup

#### Dataset

In this section, we evaluate the performance of various mitotic detection approaches on 4D microscopic images (Japan Society for Precision Engineering, Technical Committee on Industrial Application of Image Processing Appearance inspection algorithm contest 2017 (TC-IAIP A-IA2017) [34]) using a total of 16 datasets. The average size of each dataset is 480$\times$480$\times$37, and it includes 80 temporal 3D frames. Each dataset instance includes at least one and a maximum of three mitotic cells, and bounding box annotations of the mitotic cells are provided. However, the mitotic cell stages were not provided; therefore, binary classification and detection were performed in this work.

Considering the limited data and to avoid overfitting, we utilized the 2.5D method (where a target slice image and its neighbouring slices are utilized as input to extract 3D spatial information) [14] for mitotic detection rather than directly using 3D images. Each slice image (*s*) and its two neighbouring slices ($s - 1, s + 1$) are employed as one sample. We also applied data augmentation techniques to increase the size of the data, which results in improved accuracy and avoids overfitting. Image rotation, scaling, flipping, and random cropping were performed with 15$^\circ$ rotation and random scaling between 0.8 and 1.2. The total data generated from these methods augmented the original data by more than 100-fold.

#### Implementation details

We used the Adam optimizer with the initial learning rate set to $0.5 \times$ $10^{-5}$.The learning rate was changed to $10^{-7}$. Training was conducted for 60,000 iterations. In total, we set seven time sequences with three forward and backward sequences in our bidirectional CLSTM. The RDLA++ uses three consecutive slices as input to extract spatial information.

### Ablation studies

To verify the proposed network’s effectiveness, we performed ablation studies based on the CornerNet and CenterNet detection heads. The results are reported in Table 1.

**Table 1:** Results of an ablation experiment with CenterNet and CornerNet heads

Detector	Hourglass	DLA	DLA++	Multi-scale (backbone)	2.5D (spatial)	Recurrent (temporal)	Precision	Recall	F1 score
CornerNet1	X						0.0715	0.8933	0.4823
CornerNet2			X				0.1209	0.8798	0.4953
CornerNet3			X	X	X	X	0.7910	0.8778	0.8344
CenterNet1		X					0.1002	0.9018	0.5010
CenterNet2			X				0.1877	0.8822	0.5350
CenterNet3			X	X			0.2319	0.8960	0.5647
CenterNet4			X		X		0.5030	0.7511	0.6300
CenterNet5			X	X	X		0.5640	0.7409	0.65245
**CenterNet6**			X	X	X	X	0.8339	0.8752	0.8546

**Backbone.** First, to demonstrate the effectiveness of a full-scale dense connection, we compared the results of the CornerNet head using Hourglass and DLA++ and the CenterNet head using DLA and DLA++. The results of both comparisons indicate that DLA++ achieves better performances compared to the DLA and Hourglass backbones. Due to their poor performances, these methods cannot be used in real applications; their precision is less than 0.2 even for the best performances. We observed that normal cells were falsely detected and classified as mitotic cells.

**Multi-scale (backbone).** The output of DLA++ (Fig. 3c) was scaled to 4, 8, and 16; however, DLA++ was used by CenterNet2 in Table 1 only with output scale 4 when conducting the detection task. We assessed the effectiveness of multiscale output for detecting multiscale mitotic cells. Based on DLA++, we added an upsampling operation followed by a 1$\times$1 convolution and enlarged the output to scales of 16 and 8. We concatenated the 4, 8, and 16 scales and then conducted the detection task. The results are shown as CenterNet3. The multiscale output achieves enhanced performances compared to a single-scale output.

**2.5D (spatial).** Based on CenterNet2 and CenterNet3, we added an additional experiment to demonstrate the effectiveness of the spatial information (CenterNet4, CenterNet5). In this experiment, the spatial strategy was the same as that shown in Fig. 2; however, there were no GCLSTM blocks, and we used a 1$\times$1 convolution operation to merge spatial information. The 2.5D strategy enables the model to detect spatial information more accurately. The CenterNet2 results improved from 0.1877 to 0.5030 (CenterNet4), while the accuracy of CenterNet3 improved from 0.2319 to 0.5640 (CenterNet5).

**Recurrent (temporal).** To form an RDLA++ network, we used DLA++ with multiscale (backbone), 2.5D (spatial), and recurrent (temporal) components (Fig. 2). According to Table 1, RDLA++ further enhances the performance of CornerNet2 and CenterNet5 to CornerNet 3 and CenterNet6, with a precision of approximately 0.8 and an F1 score of more than 0.83. We observe that CenterNet6 achieves the best performance compared to the other state-of-the-art methods. We refer to this model as CenterNet (RDLA++) in the next two sections.

### Comparison of the state-of-the-art mitotic detection

In this section, we divided the volume data into 2D slices to assess our network performance along with those of other state-of-the-art 2D detection methods. In total, the 2D images from the slicing volume constituted approximately 3200 slices, where 1200 slices contained mitotic cells and 2000 slices contained only normal cells. To efficiently evaluate the performance of the proposed network, we compared our networks with other state-of-the-art mitotic detection methods, including SSD [16], EDCRF [35], 2D and 3D FASTER R-CNN [17], TS-BLSTM [5], SVM [36], HCNN [6], CasDetNet_CLSTM [14], a 2.5D network with 2D anchors, and CasDetNet_CLSTM_3DAnchor [15]. The performances of these networks were evaluated based on precision, recall, and F1-score metrics. A bounding box was considered correct when its IoU with the ground truth bounding box exceeded 0.6.

Table 2 represents the comparisons between DLA++, RDLA++, and other state-of-the-art methods. SVM and EDCRF are not deep learning methods, but their performances are better than some deep learning-based detection methods designed for normal image detection, such as Faster R-CNN, CornerNet, SSD, and CenterNet. These methods were designed to solve the high false positive problem of cell similarity algorithms. Faster R-CNN and CenterNet with the DLA backbone achieve high recall rates (0.93 for Faster R-CNN and 0.9 for CenterNet (DLA)); however, they have low precision-approximately 0.1 for both methods.

**Table 2:** Results of a quantitative comparison among the proposed network, non-deep learning methods, and deep learning methods

Method	Precision	Recall	F1 score
EDCRF [35]	0.6829	0.6210	0.652
SVM [36]	0.3782	0.9035	0.6409
2D Faster R-CNN [17]	0.0870	0.9310	0.509
3D Faster R-CNN	0.0592	0.4143	0.2367
SSD [16]	0.0411	0.7221	0.3816
CornerNet(Hourglass)	0.0715	0.8933	0.4823
CenterNet (DLA)	0.1002	0.9018	0.5010
HCNN [6]	0.7003	0.6910	0.6957
TS-BLSTM [5]	0.7883	0.7751	0.7817
2.5D Faster R-CNN [14]	0.3591	0.7532	0.5562
CasDetNet_CNN [14]	0.7228	0.70358	0.7132
CasDetNet_CLSTM [14]	0.8195	0.7974	0.8085
CasDetNet_3DAnchor [15]	0.8356	0.8442	0.8399
CenterNet(RDLA++)	0.8339	0.8752	0.8546

The result of Faster R-CNN with 3D convolution was used as the spatial information. However, the result was not as good as that of the original 2D Faster R-CNN because the detection model overfitted when training on the data samples (3D volume). Similar to Faster R-CNN, SSD exhibits poor performance in mitotic detection; both models generate large numbers of false positives. TS-BLSTM and HCNN were both designed to perform mitotic detection from 2D images and use motion as an extra input. These methods outperform the other methods (0.6957 and 0.7817); their main problem is that they were not designed for 4D data and do not include spatial information in their prediction. Moreover, another factor limiting the performance of TS-BLSTM is that the dataset does not include labels for the different mitosis stages. For stage refinement, we implemented the TS-BLSTM network without a bidirectional LSTM and trained the model for binary classification. CasDetNet_CLSTM, CasDetNet_CNN, and CasDetNet_CLSTM_3DAnchor were designed and applied to mitotic detection in 4D microscopic images. These CasDetNet variants achieve better detection results than do the other methods (0.71 for CasDetNet_CNN, 0.81 for CasDetNet_CLSTM, and 0.84 for CasDetNet_CLSTM_3DAnchor). Compared to the CasDetNet networks, our RDLA++ enhanced the performance of CenterNet and yielded a higher detection accuracy. The precision of RDLA++ is almost identical to that of CasDetNet_CLSTM_3Danchor, with a difference of only 0.0017, but its recall and F1-scores are higher (0.875 and 0.855, respectively).

**Fig. 5:**
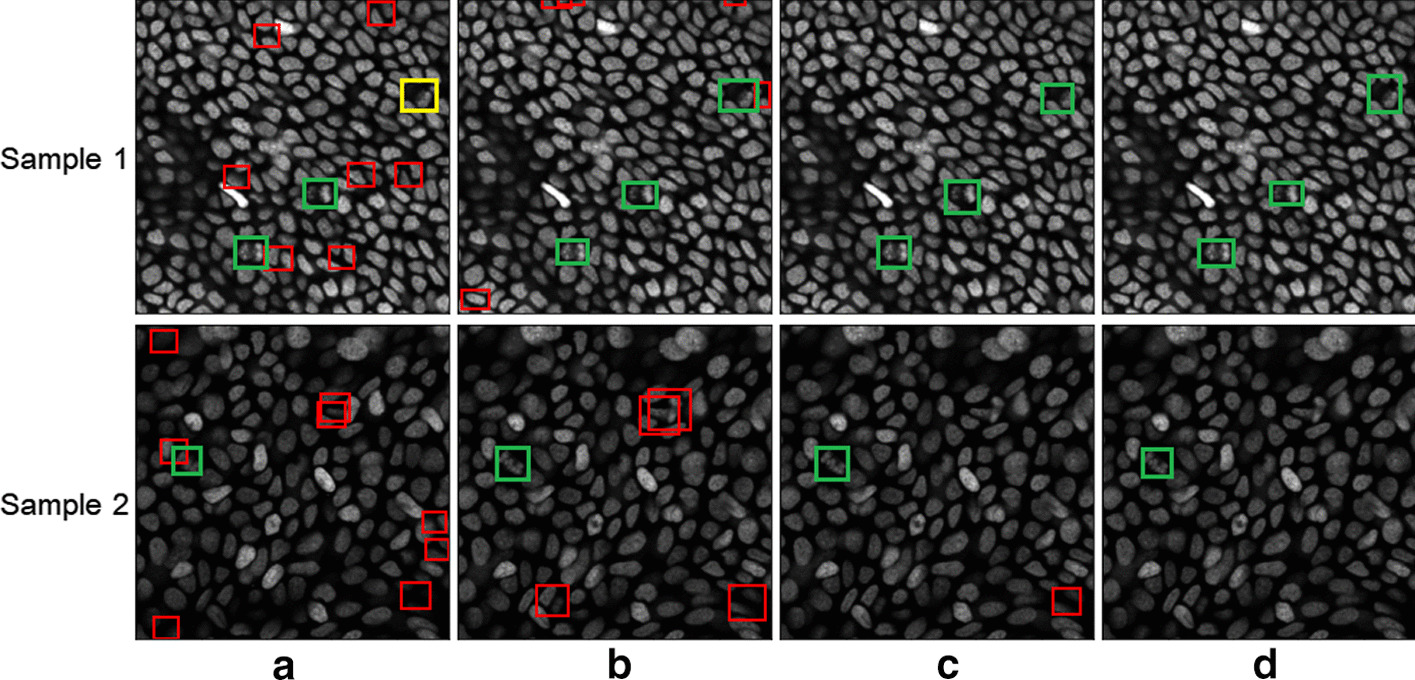
Visualization results of the detection methods for two typical microscopic slice images. **a** Faster RCNN, **b** CenterNet, **c** CenterNet (DLA++ multislice), and **d** CenterNet (RDLA++). The green bounding box represents a correct detection (TP) result, a red bounding box denotes overdetected (FP) results, and the yellow bounding box represents an underdetected (FN) result

The detection results from two typical microscopic slice images by Faster R-CNN, CenterNet, CenterNet (DLA++ multislice), and CenterNet (RDLA++) are visualized in Fig. 5a–d. The green bounding boxes represent correct detection or true positive (TP) results, the red bounding boxes denote overdetected or false positive (FP) results, and the yellow bounding boxes represent underdetected or false negative (FN) results. As shown in Fig. 5, several FPs (red bounding boxes) occur in the detection results of Faster R-CNN (Fig. 5a) and CenterNet (Fig. 5b). Moreover, Faster R-CNN (Fig. 5a) fails to detect mitotic cells and it generates FN results (the yellow bounding box) on sample 1. A comparison of the conventional CenterNet (Fig. 5b) and the proposed CenterNet with DLA++ (Fig. 5c) shows that the latter can solve the overdetection problem. Only one overdetection was found in the results (sample 2) of CenterNet with DLA++. By including temporal information, CenterNet with RDLA++ (Fig. 5d) can clearly separate mitotic cells from normal cells and mitotic cells. In the results of the proposed CenterNet with RDLA++, no FPs were detected (only mitotic cells were detected) in both sample 1 and sample 2.

### Evaluation of 4D mitotic cell detection

To evaluate the 4D detection performance, we determined the average IoU for each slice from the same region in 5 continuous slices. Average IoU scores above 0.5 were classified as true positives; otherwise, they were classified as false negatives. We considered the detection results as false positives when the average IoU from the same region in 5 continuous slices was greater than 0.5 but not with the ground truth.

In the 4D evaluation, we compared our technique with Faster RCNN, CasDetNet_CLSTM 3DAnchor, CenterNet (DLA), and Sugano (the winner of the TC-IAIP AIA 2017, which is a non-deep learning method). All approaches used IoU to calculate the 4D results except Sugano, the results of which were provided by TC-IAIP AIA 2017. Table 3 represents the methods’ results along with the results of the 4D evaluation.

**Table 3:** The detection of 4D data at 0.5 IoU to measure the orientation robustness of each method

Method	Data	1	2	3	4	5	6	7	8	9	10	11	12	13	14	15	16	Total
Sugano [37]	TP	1	1	2	3	2	1	0	1	2	2	1	1	0	2	2	2	23
	FN	0	0	0	0	1	0	1	0	0	0	1	2	1	0	0	0	6
	FP	0	3	0	0	0	0	0	0	0	9	0	0	0	1	17	6	36
Faster RCNN [17]	TP	1	1	2	3	3	1	1	1	2	2	2	3	0	2	2	2	28
	FN	0	0	0	0	1	0	0	0	0	0	0	0	1	0	0	0	1
	FP	8	5	9	12	16	4	4	7	8	7	6	9	8	4	15	11	133
CasDetNet_CLSTM [14]	TP	1	1	2	3	1	1	1	1	1	2	1	1	0	2	2	2	22
	FN	0	0	0	0	2	0	0	0	1	0	1	2	1	0	0	0	7
	FP	0	0	0	0	0	0	0	0	0	0	0	0	0	0	0	0	0
CasDetNet_CLSTM_3DAnchor [15]	TP	1	1	2	3	1	1	1	1	2	2	2	3	1	2	2	2	27
	FN	0	0	0	0	2	0	0	0	0	0	0	0	0	0	0	0	2
	FP	0	0	0	0	0	0	0	0	0	0	0	0	0	0	0	0	0
CenterNet(RDLA++)	TP	1	1	2	3	3	1	1	1	2	2	2	3	1	2	2	2	29
	FN	0	0	0	0	0	0	0	0	0	0	0	0	0	0	0	0	0
	FP	0	0	0	0	0	0	0	0	0	0	0	0	0	0	0	0	0
Ground truth		1	1	2	3	3	1	1	1	2	2	2	3	1	2	2	2	29

The Sugano method is a nondeep learning method that won the TC-IAIP AIA 2017 challenge [37]. This method has false positive problems similar to Faster R-CNN. Here, the Sugano technique is affected by the false positive problem only on samples 2, 10, 14, 15, and 16; however, Faster R-CNN exhibits the false positive problem for all the data. Samples 11, 12, and 13 include a problematic orientation of mitotic cells. Consequently, these cells are not detected by most of the other methods except for CasDetNet_CLSTM.

The example results (case 5) of CasDetNet_CLSTM_ 3DAnchor [15] and CenterNet (RDLA++) are shown in Table 3. The two mitotic cells were not detected by CasDetNet_CLSTM_3DAnchor [15]; however, they were detected perfectly by the proposed method. In addition to accurate detection, the proposed technique detects mitotic cells faster than does CasDetNet_CLSTM_3DAnchor [15]. Table 4 shows a computation time comparison. The computation time of the proposed method with postprocessing is 1.8 times faster than that of CasDetNet_CLSTM_3DAnchor when both are run on 1 GPU. Moreover, the computation time of the proposed method running on 1 GPU was nearly the same as that of CasDetNet_CLSTM_3DAnchor running on 4 GPUs. When considering only detection time (without postprocessing), the proposed method performs up to 3 times faster than CasDetNet_CLSTM_3DAnchor running 4 GPUs and 9 times faster CasDetNet_CLSTM_3DAnchor running on 1 GPU.

**Table 4:** Detection time comparison (ms/slice)

Method	Detection time
CasDetNet (1 GPU) [15]	4641
CasDetNet (4 GPUs) [15]	2412
CenterNet(RDLA++) (1 GPU)	2533

## Discussion

As shown in Table 2, machine learning and shallow learning models (SVM and EDCRF) can show better results than do the conventional deep learning methods proposed to perform detection tasks using normal images. This result occurs for two reasons. First, deep learning-based detection methods require large numbers of training samples, while the shallow learning models do not require such large numbers of training samples. In this research, the number of training samples is limited to 16, which may significantly decrease the performances of existing deep learning-based approaches designed for 2D image detection. Second, some shallow learning models such as EDCRE are designed specifically for mitotic tasks with temporal information, while the conventional deep learning-based methods (i.e., Faster R-CNN, SSD, CenterNet, and CornerNet) are designed for 2D image detection, and they extract 2D spatial features without temporal information. We observed that the deep learning methods designed for mitotic cell detection (HCNN, CasDetNet CLSTM and TS BLSTM) yield better performances and that all these networks consider temporal information. These experiments demonstrate that temporal information is important for the mitotic detection task.

Compared to 2D Faster R-CNN (which utilizes only a target 2D slice image as input) and 3D Faster R-CNN (which uses a 3D volume as input), 2.5D Faster R-CNN (which takes the target slice image and its neighbouring slices as input) achieved a better performance. This result occurs because the 2.5D CNN is able to extract 3D spatial information while the 2D CNN cannot extract 3D information, which results in lower precision. Moreover, although a 3D CNN can extract 3D spatial information, it includes more parameters and requires several 3D volumes for training; thus, the performance of 3D Faster R-CNN was significantly degraded by the limited data. Therefore, the 2.5D method is important for mitotic detection in 4D microscopic images.

According to the experimental results, the performance of our proposed method exceeds that of other state-of-the-art methods in terms of both computation time and detection accuracy. The 4D mitotic cell detection of CasDetNet CLSTM 3DAnchor and our network may be identical when they are evaluated on the same region of 5 continuous slices (> 0.7 of IoU); however, by reducing the average IoU to 0.5 with 5 continuous slices, our method solves the problem of false negatives in the data. Nevertheless, reducing the IoU threshold is not the best technique to obtain satisfactory results. Thus, there is still a need to improve the performance of the network because it achieves insufficient detection results for some data.

## Conclusion

In this study, we proposed a full-scale connected recurrent deep layer aggregation (RDLA++) network for mitotic detection from 4D microscopic images. The proposed dense level skip connections (DLA++) are utilized to improve the scale features and reduce the network parameters. The network performs more efficiently due to its feature reuse. The recurrent connections are designed to extract temporal and spatial information from 4D data and integrate them into the 2.5D concept. With DLA++ and RDLA++, the scale, temporal, and spatial features are enhanced to improve the detection accuracy. Both RDLA++ and DLA++ can be integrated with any detection head from as state-of-the-art anchor-free method such as CornerNet or CenterNet. The limitation of our method is that it requires a 4D dataset for network execution a 3D volume and time sequences. Without the 4D information, the performance of our method is decreases drastically. The other methods that are considered in our experiments are designed for 2D or 3D datasets. These methods perform mitotic detection either without spatial information from a 3D volume or without temporal information from time sequences.

## Data Availability

The datasets generated and/or analysed during the current study are available in ViEW contest, http://alcon.itlab.org/18/.
